# A Rare Case of Hip Pain Secondary to Pigmented Villonodular Synovitis

**DOI:** 10.5811/cpcem.2019.12.45253

**Published:** 2020-02-24

**Authors:** Gary Lai, Brett Staller, Bhaskar Ganguly, Quan Ta, Alexander J. Scumpia

**Affiliations:** * Broward Health, Department of Emergency Medicine, Coral Springs, Florida; † Broward Health, Department of Diagnostic Radiology, Coral Springs, Florida; ‡ Broward Health, Department of Internal Medicine, Coral Springs, Florida; § Florida Atlantic University, Boca Raton, Florida

## Abstract

A 19-year-old Asian male presented to our emergency department with atraumatic right hip pain radiating to the right groin associated with pain on ambulation. Magnetic resonance imaging of the right hip with and without contrast revealed the diagnosis. Pigmented villonodular synovitis is a rare, monoarticular benign tumor originating from the synovium of the joint. The treatment is synovectomy of the pathological joint to prevent further disease progression.

## CASE PRESENTATION

A 19-year-old Asian male presented to our emergency department (ED) with a one-day history of atraumatic right hip pain radiating to the right groin associated with pain on ambulation. The patient denied weakness or numbness of his extremity, fever, chills, or recent illness. Physical examination only revealed decreased range of motion and pain with internal rotation and flexion of the right hip. ED labs consisting of complete blood count, complete metabolic profile, C-reactive protein, and erythrocyte sedimentation rate were within normal limits. Ultrasound with Doppler of the patient’s scrotum was unremarkable. Computed tomography of the abdomen and pelvis with intravenous contrast demonstrated a right hip fluid collection consistent with inflammation or infection. Magnetic resonance imaging (MRI) of the right hip with and without contrast suggested the diagnosis (Image).

## DISCUSSION

### Pigmented villonodular synovitis

Pigmented villonodular synovitis (PVNS) is a rare, monoarticular benign tumor originating from the synovium of the joint.^1^,^2^ More commonly, this tumor is slow-growing, involving a localized portion of the joint or, in rarer cases, diffuse with malignant-type features (ie, involving the entire joint, or extra-articular lesions).^3^–^5^ The incidence of intra-articular PVNS predominately occurs in young adults (median age of 30 years) and has been reported to be 1.8 per million with equal gender distribution.^4^ The hip is the second most common joint affected (15% of all cases), with the knee the most prevalent of joints affected. MRI is the radiographic study of choice to identify hyperplastic synovium lesion(s) characteristic of PVNS.^4^ The treatment is complete synovectomy of the pathological joint to prevent further disease progression; with a recurrence rate of 7.7 to 17.8%.^1^ This case illustrates the necessity of a broad ED differential diagnosis (i.e., neoplasms, infection, etc.) accompanied with multiple diagnostic modalities for optimum patient outcome.

**CPC-EM Capsule**

What do we already know about this clinical entity?

Pigmented villonodular synovitis (PVNS) is a rare, monoarticular benign tumor originating from the synovium of the joint.

What is the major impact of the image(s)?

The image demonstrates the hypointense lesion caused by the hemosiderin deposition in the hyperplastic synovium characteristic of PVNS.

How might this improve emergency medicine practice?

This case illustrates the necessity of a broad differential diagnosis in a very common patient chief complaint (arthralgia) for optimum patient outcome.

## Figures and Tables

**Image:**
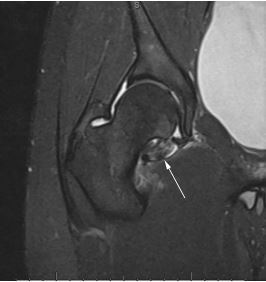
Coronal magnetic resonance imaging (T1-weighted) of the patient’s right hip demonstrating the hypointense lesion caused by the hemosiderin deposition in the hyperplastic synovium characteristic of pigmented villonodular synovitis (white arrow).
